# Topological Nodal States in Circuit Lattice

**DOI:** 10.1155/2018/6793752

**Published:** 2018-09-02

**Authors:** Kaifa Luo, Rui Yu, Hongming Weng

**Affiliations:** ^1^School of Physics and Technology, Wuhan University, Wuhan 430072, China; ^2^Beijing National Laboratory for Condensed Matter Physics, Institute of Physics, Chinese Academy of Sciences, Beijing 100190, China

## Abstract

The search for artificial structure with tunable topological properties is an interesting research direction of today's topological physics. Here, we introduce a scheme to realize topological nodal states with a three-dimensional periodic inductor-capacitor (LC) circuit lattice, where the topological nodal line state and Weyl state can be achieved by tuning the parameters of inductors and capacitors. A tight-binding-like model is derived to analyze the topological properties of the LC circuit lattice. The key characters of the topological states, such as the drumhead-like surface bands for nodal line state and the Fermi arc-like surface bands for Weyl state, are found in these systems. We also show that the Weyl points are stable with the fabrication errors of electric devices.

## 1. Introduction

Recently, there is great interest in realizing topological states in various platforms. Topological states, including the quantum Hall states, quantum spin Hall states, Dirac states, Weyl states, and nodal line states, have achieved significant progresses in electronic materials [1–12], cold atoms [13–22], photonics [23–29], phononics [30–36], and mechanical systems [37–47]. In addition, the topological properties in electric circuit system have also been explored in several works [48–53]. The quantum spin Hall-like states have been proposed in two-dimensional circuit lattice via time-reversal symmetric Hofstadter model [48, 49]. The Weyl state has been found in three-dimensional circuit network and proposed to be able to be detected from the boundary resonant signal [51]. The topological Zak phase is discussed in the one-dimensional SSH-type circuit lattice [52]. These proposed electric circuits are composed of interconnected linear lossless passive elements, such as capacitors and inductors. The significant advantages of the circuit lattice are that the parameters of the system are independently artificial adjustable and the symmetry of the lattice is protected by the parameters of electronic components and the way they are connected, rather than their positions in the real space.

From symmetry considerations, three types of nodal line states have been proposed in a great number of literatures [54–56]. Type-A is protected by mirror reflection symmetry [28, 57–61], type-B is protected by the coexistence of time-reversal and space inversion symmetry [20, 21, 59, 62–72], and type-C is protected by the nonsymmorphic space group [73–76]. While the existence of Weyl points does not require any crystalline symmetries except the lattice translational symmetry, Weyl points can only appear when the bands degeneracy is removed by breaking either time-reversal [3, 4] or spacial inversion symmetry [5, 6] as summarized in the review articles [10–12]. In the present work, we demonstrate a feasible strategy to design both nodal line state and Weyl state in a three-dimensional circuit lattice. The nodal line structure we obtained belongs to type-B as discussed above. The Weyl state is achieved by breaking the spacial inversion symmetry. The topological phase transition between them can be controlled by tuning the parameters of the components. In order to investigate their topological properties, we transform the circuit network problem to a tight-binding-like model. Based on the tight-binding model, the novel surface states, including drumhead-like surface bands for nodal line state and Fermi arc-like surface bands for Weyl state, are found in the surface of the circuit lattices. Moreover, the stability of the Weyl points under perturbation of fabrication errors is studied.

## 2. Results

### 2.1. Models and Theoretical Framework

**Figure 1:**
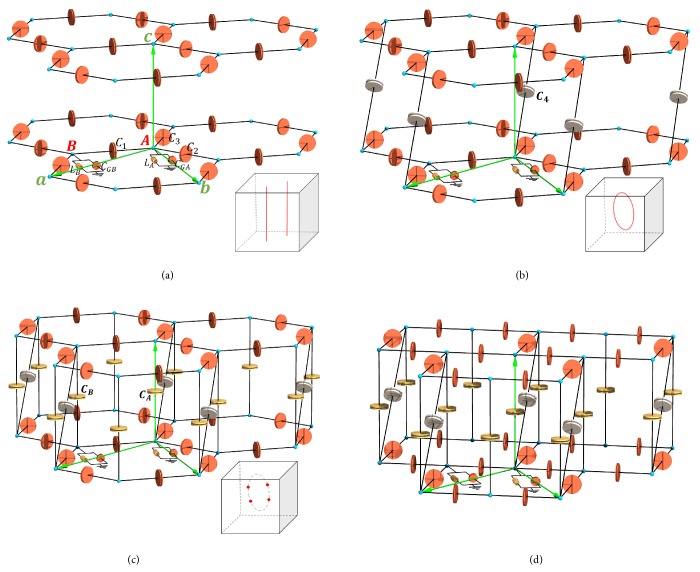
Schematic setup of the three-dimensional LC circuit lattice. (a) Honeycomb layers consisting of inductors and capacitors stack along **c** direction without connection between each layer. The primitive unit cell consists of two inequivalent nodes A and B, which are linked by capacitors *C*_1_, *C*_2_, and *C*_3_ in the **a**-**b** plane. Each node *A* (*B*) is grounded through the parallel connected inductor *L*_*A*_ (*L*_*B*_) and capacitor *C*_*GA*_ (*C*_*GB*_). Lattice vectors are denoted as **a**, **b**, and **c**. The frequency bands structure of the single layer honeycomb LC lattice has two band-crossing points, which are uniform in **k**_**c**_ direction and form two straight nodal lines in the BZ (red colour lines in the inset). (b) Connecting nodes A and nodes B between neighbor-layers with *C*_4_. The nodal lines become **k**_*c*_ dependent and form a closed ring by choosing appropriate *C*_4_. (c) Connecting nodes A-A and nodes B-B between neighbor-layers with *C*_*A*_ and *C*_*B*_, respectively, and removing the space inversion symmetry by tuning *C*_*A*_ ≠ *C*_*B*_ and *C*_*GA*_ ≠ *C*_*GB*_, the nodal ring may be degenerated to Weyl points. The LC lattice can be deformed into (d), which brings convenience for constructing circuit elements in experiments with spectrum topologically invariable.

The design scheme of realizing the nodal line state and Weyl state in LC circuit lattice is shown below. We consider a honeycomb lattice consisting of capacitors and inductors in **a**-**b** plane as a starting point. The subnodes A and B are linked by capacitors *C*_1_, *C*_2_, and *C*_3_. Every node *A* (*B*) is grounded through the parallel connected inductor *L*_*A*_ (*L*_*B*_) and capacitor *C*_*GA*_ (*C*_*GB*_) as shown in Figure 1(a). Similar to the electronic band structure of graphene, the frequency spectrum of the single layer LC honeycomb lattice contains two band-crossing points in the two-dimensional Brillouin zone (BZ). Stacking the two-dimensional honeycomb lattice along **c** direction without any coupling between each other, the band-crossing points will form two straight nodal lines in the three-dimensional BZ as shown in Figure 1(a). In order to make the straight nodal lines **k**_*c*_ dependent, we connect nodes A and B between neighbor-layers with *C*_4_ as shown in Figure 1(b). By tuning the value of *C*_4_, these two separate lines are deformed and merged into a closed ring, namely, the nodal line we are searching for. The nodal line structure is protected by the coexistence of time-reversal and space inversion symmetry. If the space inversion symmetry of the circuit system is removed, the continuous nodal line may be degenerated to discrete Weyl points [25]. Following this insight, nodes A-A and B-B between the nearest-neighbor-layers are connected with *C*_*A*_ and *C*_*B*_, respectively, as shown in Figure 1(c). *C*_*A*_ and *C*_*B*_ and *C*_*GA*_ and *C*_*GB*_ are deliberately set to be different, resulting in the space inversion symmetry breaking and emergence of the Weyl points. The LC circuit lattice in Figure 1(c) can be deformed to Figure 1(d). The latter is more convenient to construction of circuit elements in experiments with spectrum topologically invariable. The band structures and the topological properties of the nodal line state and Weyl state for lattice given in Figures 1(b), 1(c), and 1(d) will be detailed in the remain text.

Here, we study the resonance condition of the circuit lattice, where a nonzero distribution of potential satisfies Kirchhoff's law. We follow the method given in Ref. [77], where the periodical circuit lattice problem is transformed into a tight-binding-like model in the momentum space. This approach relies on an analogy between Kirchhoff current equation in periodic circuit lattice and the quantum mechanics with periodic crystalline structure. Therefore, the circuit band structure arises in a manner analogous to electronic band structure in crystals. The tight-binding-like model for the circuit lattice in Figures 1(c) and 1(d) is given as

(1) $$\begin{array}{c} H\left(\;k\;\right)|\;V\left(\;k\;\right)\;\rangle =\frac{1}{\omega^{2}L}|\;V\left(\;k\;\right)\;\rangle , \end{array}$$

where

(2) $$\begin{array}{c} H\left(\;k\;\right)=\overset{3}{\underset{i=0}{\sum }}d_{i}\left(\;k\;\right)\sigma_{i}, \end{array}$$

*ω* is the resonance frequency of the circuit lattice, matrix *L* = diag⁡(*L*_*A*_, *L*_*B*_), *𝒱* = (*𝒱*_*A*_, *𝒱*_*B*_)^*T*^ is the Bloch states for the potential distributions, and the Pauli matrices are for the space spanned by (*𝒱*_*A*_, *𝒱*_*B*_). The coefficients in front of the Pauli matrices are given as

(3) d0k=C1+C2+C3+C4+CGA+CGB2+CA+CB1−cos⁡kc,

(4) d1k=−C1−C2cos⁡kb−ka−C3cos⁡ka−C4cos⁡kc−ka,

(5) d2k=C2sin⁡kb−ka−C3sin⁡ka+C4sin⁡kc−ka,

(6) d3k=CGA−CGB2+CA−CB1−cos⁡kc.

To simplify the calculations without loss of generality, we set *L*_*A*_ = *L*_*B*_ = *L*_*G*_; therefore the matrix *L* in (1) is proportional to an identity matrix. Solving (1), we obtain two branches of dispersions $\omega_{1,2}^{-2}(k)=L_{G}\left[d_{0}(k)\pm \sqrt{d_{1}^{2}(k)+d_{2}^{2}(k)+d_{3}^{2}(k)}\right]$ in the BZ. By varying the parameters of capacitor *C*_*i*_s, we can artificially tune the dispersion of *ω*_1,2_^−2^(**k**) and obtain the desired bands structure. In the following section, we will show that the nodal ring-type and Weyl point-type bands touching points are available in our circuit lattice.

### 2.2. Nodal Line and Drumhead-Like Surface State

**Figure 2:**
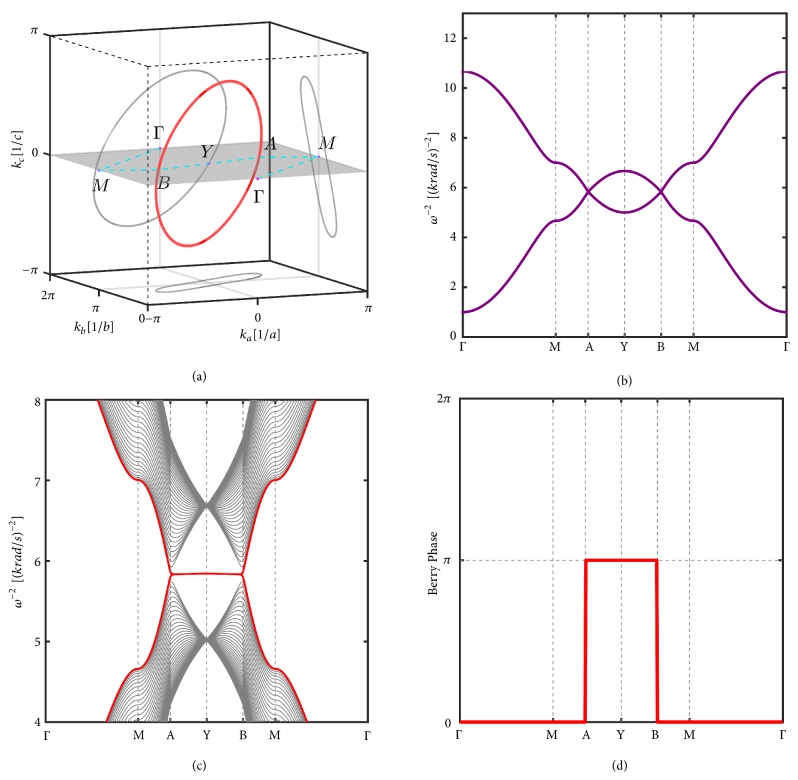
(a) Nodal line (in red) in the BZ and its projection on the (001), (010), and (100) planes (in grey). The parameters are set as *C*_1_ = 1*mF*, *C*_2_ = 2*mF*, *C*_3_ = 1*mF*, *C*_4_ = 0.833*mF*, *C*_*GA*_ = *C*_*GB*_ = 1*mF*, and *L*_*A*_ = *L*_*B*_ = 1*mH*. (b) Band structure along Γ − *X* − *A* − *B* − *X* − Γ, where A and B are two points with *k*_*c*_ = 0 on the nodal line as labeled in (a). (c) The band dispersions with the surface states (red colour lines) on the (001) surface. The drumhead-like surface states are nestled inside the projection of the nodal ring. (d) The Berry phase *θ*_*k*_||__ equals *π* for *k*_||_ inside the nodal ring, while it is zero for *k*_||_ outside the nodal ring.

The closed loops of bands crossing points can be protected by the coexistence of space inversion symmetry and time-reversal symmetry [25]. The LC circuit is time-reversal symmetric by nature; we only need to choose a group of parameters of the inductors and capacitors to preserve the space inversion symmetry. For the circuit network given in Figure 1(b), the space inversion symmetry can be obtained by setting *C*_*GA*_ = *C*_*GB*_, with the inversion center located at the middle point of node A and node B. In this case, *d*_3_(**k**) vanishes (*C*_*A*_ = *C*_*B*_ = 0 in Figure 1(b)). The conditions for bands degeneracy require *d*_1_(**k**) = 0 and *d*_2_(**k**) = 0 to be satisfied simultaneously, leading to two restrictions for three variables **k** = (*k*_*a*_, *k*_*b*_, *k*_*c*_), which gives one-dimensional solution space and forms a continuous nodal ring in the **k** space.  Figure 2(a) illustrates obtained nodal ring structure centered at *Y* = (0, *π*, 0) point. The band dispersions along Γ-*M*-*A*-*Y*-*B*-*M*-Γ are shown in Figure 2(b), where A and B are two nodal points in *k*_*c*_ = 0 plane.

The topological properties of a nodal line can be inferred from the winding number (−1)^*ζ*_1_^ = (1/*π*)∮_*C*_*𝒜*(**k**) · *d ***k**, where *𝒜*(**k**) is the Berry connection and *C* is a closed loop in the momentum space pierced by the nodal line. *ζ*_1_ = 1 in our model means that the nodal line is topologically stable. Due to the bulk-boundary correspondence, the nontrivial nodal line structure indicates a novel surface state. Based on the tight-binding-like model 2, the surface state in the (001) direction is calculated as shown in Figure 2(c), where a flat surface band nestles inside of the projected node-ring. In order to relate the nontrivial topology induced by the bulk band singularity to edge modes, we calculate the Berry phase of the one-dimensional systems *H*_*k*_||__(*k*_*c*_) parameterized by the in-plane momentum *k*_||_ = (*k*_*a*_, *k*_*b*_). The Berry phase for the one-dimensional system is defined as *θ*_*k*_||__ = ∫*𝒜*_*k*_||__*dk*_⊥_, where *𝒜*_*k*_||__ is the Berry connection matrix defined as *𝒜*_*k*_||__ = 〈*𝒱*_1_|(**k**)∣*i*∂_*k*_*c*__|*𝒱*_1_(**k**)〉. In the one-dimensional parametrized systems, the Berry phase equals *π* for *k*_||_ inside the nodal ring, while it is zero for *k*_||_ outside the nodal ring as shown in Figure 2(d).

### 2.3. Weyl Points and Surface ‘Fermi Arc'

**Figure 3:**
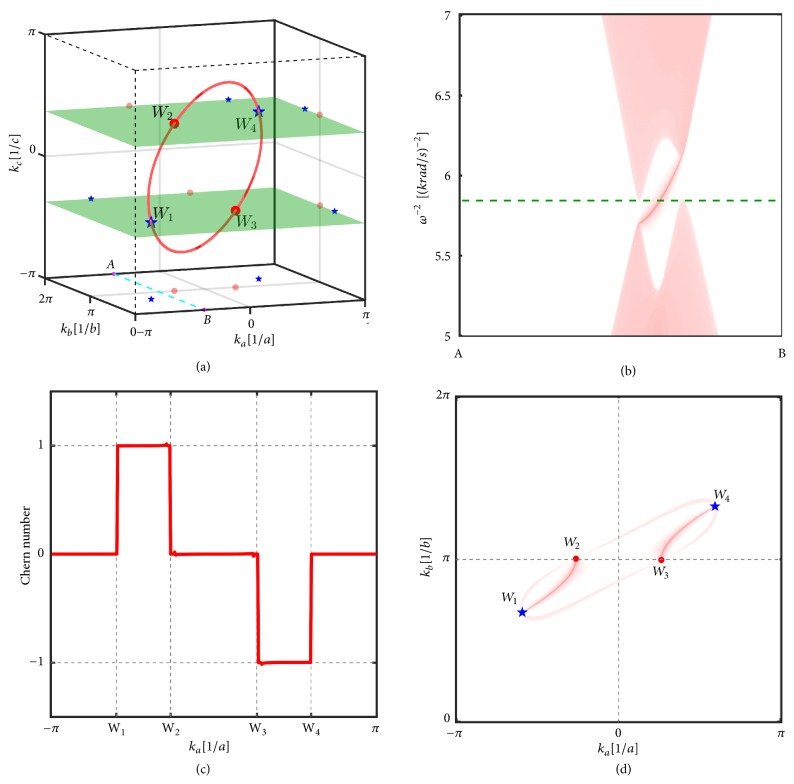
(a) Four Weyl points in the Brillouin zone and their projections on (001), (010), and (100) direction. *C*_*A*_ = 0.2*mF*, *C*_*B*_ = 0.01*mF*, and *C*_*GA*_ = 0.77*mF* are used in the calculations. The other parameters are the same as Figure 2. The positions of the Weyl points are the intersection points between the nodal ring determined by *d*_1_(**k**) = *d*_2_(**k**) = 0 and the two planes determined by *d*_3_(**k**) = 0. The chirality are indicated as blue stars for *χ* = +1 and red points for *χ* = −1. (b) The gapless surface band dispersions on the A-B-*k*_*c*_ plane and terminates in the (001) direction. (c) The Chern numbers for the two-dimensional planes perpendicular to *k*_*a*_. Moving along *k*_*a*_, the Chern number increases (decreases) when the plane passing through the Weyl points with +1  (−1) chirality. (d) On the (001) surface, Fermi arcs connect the projections of the bulk Weyl nodes of opposite chiralities onto the surface.

Starting with nodal line states, Dirac states or Weyl states are possible to emerge by introducing symmetry breaking terms [25]. Here we show the Weyl states can be realized in the circuit lattice shown in Figures 1(c) and 1(d). The space inversion symmetry of the circuit lattice is easy to be removed by setting *C*_*GA*_ ≠ *C*_*GB*_ and *C*_*A*_ ≠ *C*_*B*_. In this case, *d*_1_(**k**) and *d*_2_(**k**) are not affected and the nodal ring structure as solutions of *d*_1_(**k**) = *d*_2_(**k**) = 0 remains. Now the gap closing condition further requires *d*_3_(**k**) = 0, which leads to cos⁡*k*_*c*_ = 1 + (*C*_*GA*_ − *C*_*GB*_)/2(*C*_*A*_ − *C*_*B*_). Tuning the parameters of *C*_*GA*_, *C*_*GB*_, *C*_*A*_, and *C*_*B*_ to make the absolute value of the right-hand side of the equation less than 1, *d*_3_(**k**) = 0 determines two planes perpendicular to *k*_*c*_ axis in the first BZ. When these two planes intersect with the nodal ring, as shown in Figure 3(a), there are four crossing points that are the desired Weyl points in the Brillouin zone. Because time-reversal symmetry maps Weyl point at **k** to −**k** with the same chirality, to neutralize the chirality in the BZ there must be two other Weyl points with opposite chirality. Therefore the minimal number of Weyl points in our circuit lattice has to be four. The chiralities of the Weyl points are determined in the following way. We calculate the Chern number for the lower bulk bands on the planes perpendicular to *k*_*a*_ axis in the 3D BZ. As shown in Figure 3(c), moving along *k*_*a*_, the increasing (decreasing) of Chern number when the plane passes through the Weyl points indicates that the chiral value of the Weyl points is +1  (−1). The Weyl points with +1 chirality are marked with blue star while red points are for −1 chirality in Figure 3(a).

As a result of the nonzero Chern numbers, topologically protected gapless chiral surface states emerge in the band gap away from the Weyl points. An example of the nontrivial surface band dispersions is shown in Figure 3(b). A surface mode profile at the frequency of the Weyl points is also shown in Figure 3(d). Similar to the Fermi arcs in Weyl semimetals, Weyl pairs with opposite chirality are connected through the segment-like surface dispersion in the surface Brillouin zone. The surface “Fermi arc” for (100) and (010) directions surface can be calculated with the similar method.

### 2.4. Stability of the Weyl Points

**Figure 4:**
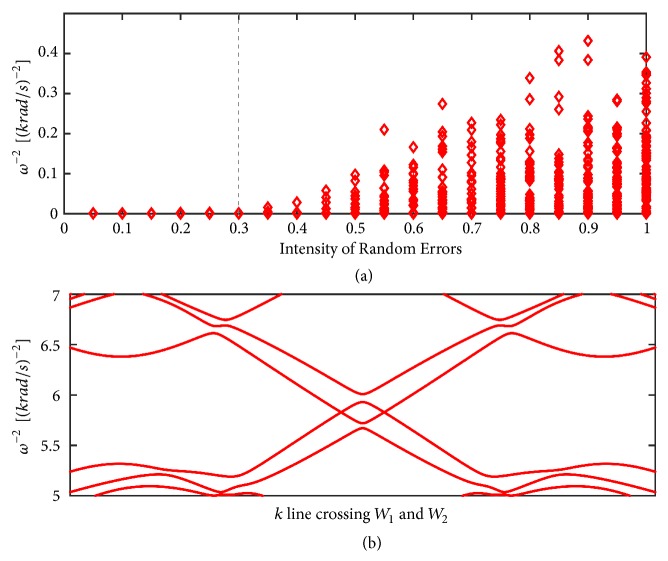
(a) The band gap, min⁡[*ω*_*N*/2+1_^−2^ − *ω*_*N*/2_^−2^] as a function of the tolerance values for a 3 × 3 × 3 super cell. *N* is number of total bands for the 3 × 3 × 3 super cell. The parameters are the same as Figure 3 without considering the fabrication errors. After taking the fabrication errors into consideration, we repeat 100 times calculations for each tolerance values. The numerical results show that the gap equals zero; i.e., Weyl points exist for the tolerance values less than 30%. Exceeding this value, the gap opens by the fabrication errors. (b) The band dispersions along the **k** line crossing two Weyl points for a 3 × 3 × 3 super cell with ±20% tolerance values on the capacitors.

In the above discussions, the inductance and capacitance in the circuit lattice are proposed as a set of precise values, but the fabrication error of the electronic devices brings about certain range of tolerance values. In this paragraph, we discuss whether the fabrication errors influence the results given above. For the nodal line state, maintaining intrinsic space inversion symmetry is a necessary condition. When the fabrication errors are taken into consideration, intrinsic space inversion symmetry is too demanding to preserve; therefore nodal line becomes unstable. However, the existence of the Weyl points does not require extra symmetries except the discrete translation invariant symmetry. It is expected to be more stable than the nodal line state under the perturbation of the fabrication errors. In order to investigate the stability of the Weyl points in the circuit lattice, we employ a 3 × 3 × 3 super cell. In addition, the values of the capacitors' parameters are randomly taken within a certain range around the precise values. In the language of solid state physics, the hopping terms and on-site energies in (6)-(9) are randomly taken. The details are shown in the materials and methods part. To simplify the calculation without loss of key points, we keep *L*_*A*_ and *L*_*B*_ fixed. For each value, we repeat calculations 100 times with different random errors and find the minimal value of the gap between band *N*/2 + 1 and *N*/2 in each time. Here, *N* is the number of total bands for the 3 × 3 × 3 super cell. As long as these two bands are not gaped, i.e., the gap is zero, the Weyl points still survive. The numerical results in Figure 4(a) show that the Weyl points exist for tolerance values around the precise value less than 30%. Exceeding this tolerance value, the gap opens and we get a topologically trivial circuit. Take a super cell with ±20% tolerance values, for example, we calculate its band structures along the **k** line crossing two of the gap closing points. The band dispersions in Figure 4(b) show that two bands cross each other and form a pair of Weyl points near *ω*^−2^ = 5.8(*krad*/*s*)^−2^.

## 3. Discussion

To summarize, we report that the topological nodal line state and Weyl state can be realized in a three-dimensional classical circuit system. We derived a two-by-two tight-binding-like model to investigate its topological nature. Based on this model, we show that the nodal line structure is protected by the inversion symmetry, which can be achieved by setting *C*_*GA*_ = *C*_*GB*_ and *C*_*A*_ = *C*_*B*_. When the inversion symmetry is broken, gap opens along the nodal line, while four Weyl points are left in the first BZ. We also confirm that the Weyl state is robust with the fabrication error of the electrical devices. The key characters of the above two topological state, namely, the drumhead-like surface states for nodal line state and ‘Fermi arcs' surface state for Weyl state, are conformed by the tight-binding-like model terminated on the (001) direction surface. The topological phase transition between nodal line state and Weyl state can be tuned by changing the parameters of the capacitors and inductors or by controlling the connecting or disconnecting of some elements, for example, the capacitors *C*_*A*,*B*_ and *C*_*GA*,*GB*_ in Figure 1(c), in the circuit lattice. Moreover, in the experimental aspect, the proposed circuit lattice can be manufactured with current technology. The topological properties, such as the patterns for the “Fermi arcs” connecting the Weyl point pairs, can be detected by measuring the potential distribution on the surface of the circuit lattice. This work offers a new, robust platform for realizing and tuning topological nodal line state and Weyl state in the classical system.

## 4. Materials and Methods

In this section, we present the details of the methods used to calculate the band structure of the circuit lattice. We start with deriving the tight-binding-like model given in (1). The current *I* passing through a two-terminal circuit element for a given drop in voltage Δ*v* is described by Ohm's law *I* = *Y*Δ*v*, where *Y* is admittance. In an alternating current (AC) sinusoidal circuits, the admittance for the ideal inductors and capacitors is *Y*_*L*_ = 1/*jωL* and *Y*_*C*_ = *jωC*, respectively. *ω* is the frequency for the sinusoidal signal and $j=\sqrt{-1}$, following the convention in electric circuits. The currents flow into nodes A and B in the cell located at **R** = 0 of circuit lattices given in Figures 1(c) and 1(d) of main text are given as

(7) IA0=jωC1vB0−vA0+C2vBb−a−vA0+C3vB−a−vA0+C4vBc−a−vA0+CAvAc−vA0+CAvA−c−vA0+CGA0−vA0+0−vA0jωLA=jωC1vB0+C2vBb−a+C3vB−a+C4vBc−a+CAvAc+CAvA−c−CGA+C1+C2+C3+C4+2CAvA0−vA0jωLA,

and

(8) IB0=jωC1vA0−vB0+C2vAa−b−vB0+C3vAa−vB0+C4vAa−c−vB0+CBvBc−vB0+CBvB−c−vB0+CGB0−vB0+0−vB0jωLB=jωC1vA0+C2vAa−b+C3vAa+C4vAa−c+CBvB−c+CBvBc−CGB+C1+C2+C3+C4+2CBvB0−vB0jωLB.

Kirchhoff's current law demands that *I*_*A*_(0) and *I*_*B*_(0) are zero. Dividing *jω* on both sides of (7) and (8), we get

(9) −C1vB0+C2vBb−a+C3vB−a+C4vBc−a+CAvAc+CAvA−c−CGA+C1+C2+C3+C4+2CAvA0=1ω2LAvA0,

(10) −C1vA0+C2vAa−b+C3vA−b+C4vAa−c+CBvB−c+CBvBc−CGB+C1+C2+C3+C4+2CBvB0=1ω2LBvB0.

With the similar method, we can obtain the equations for the potential distribution *v*_*A*_(**R**) and *v*_*B*_(**R**) on the whole lattice. Writing these equations in a matrix form, we have

(11) $$\begin{array}{c} YV=\frac{1}{\omega^{2}L}V, \end{array}$$

where *V* is a vector for the potential distribution on A and B nodes of the circuit lattice, *L* is a diagonal matrix composed of *L*_*A*_ and *L*_*B*_, and *Y* is the admittance matrix containing all information of the involving capacitors in our circuit. Equation (11) is an eigenvalue problem, in which *V* is the wave function and 1/*ω*^2^*L* is the eigenvalue. Following this insight, the admittance matrix *Y* can be interpreted as tight-binding Hamiltonian in real space. Hopping terms and on-site energies of the tight-binding model can be extracted from (9) and (10), which are listed below:

(12) HABR=0=−C1;HABR=b−a=−C2;HABR=−a=−C3;

(13) HABR=c−a=−C4;HAAR=±c=−CA;HBBR=±c=−CB;

(14) HAAR=0=CGA+C1+C2+C3+C4+2CA;

(15) HBBR=0=CGB+C1+C2+C3+C4+2CB,

where **R** is the lattice vector and *H*_*mn*_(**R**) are the tight-binding parameters between node *m* located at the home unit cell and node *n* located at **R**. With these terms, the tight-bind Hamiltonian in the momentum **k** space can be obtained through Fourier transformation *H*_*mn*_(**k**) = ∑_**R**_*e*^*i ***k**·**R**^*H*_*mn*_(**R**). The elements of the Hamiltonian are given as

(16) HAAk=CGA+C1+C2+C3+C4+2CA−CAeikc+e−ikc,

(17) HBBk=CGB+C1+C2+C3+C4+2CB−CBeikc+e−ikc,

(18) HABk=−C1−C2eikb−ka−C3e−ika−C4eikc−ka,

with the Bloch-like basis function |*𝒱*_*m*_(**k**)〉 = ∑_**R**_*e*^*i ***k**·**R**^ | *v*_*m*_(**R**)〉, *m* = *A*, *B*. Rewriting the 2 by 2 Hamiltonian matrix with the help of Pauli matrices, we get

(19) $$\begin{array}{c} H\left(\;k\;\right)=\overset{3}{\underset{i=0}{\sum }}d_{i}\left(\;k\;\right)\sigma_{i} \end{array}$$

with

(20) d0k=C1+C2+C3+C4+CGA+CGB2+CA+CB1−cos⁡kc,

(21) d1k=−C1−C2cos⁡kb−ka−C3cos⁡ka−C4cos⁡kc−ka,

(22) d2k=C2sin⁡kb−ka−C3sin⁡ka+C4sin⁡kc−ka,

(23) d3k=CGA−CGB2+CA−CB1−cos⁡kc,

which is the tight-binding-like model given in (2).

For the single layer honeycomb circuit lattice given in Figure 1(a), *C*_4_, *C*_*A*_, and *C*_*B*_ vanish. If we set *C*_*GA*_ = *C*_*GB*_, we have

(24) d1k=−C1−C2cos⁡kb−ka−C3cos⁡ka,

(25) d2k=C2sin⁡kb−ka−C3sin⁡ka,

and

(26) $$\begin{array}{c} d_{3}\left(\;k\;\right)=0. \end{array}$$

The appearance of gap closing points requires *d*_1_(**k**) = *d*_2_(**k**) = 0, which lead to

(27) $$\begin{array}{c} C_{1}+C_{2}e^{-i\left(k_{a}-k_{b}\right)}+C_{3}e^{-ik_{a}}=0. \end{array}$$

As long as the three values of *C*_1_, *C*_2_, and *C*_3_ satisfy the triangle inequality theorem, two band-crossing points can be found in the *k*_*a*_-*k*_*b*_ plane.
